# CRISPR/Cas9-mediated genome editing reveals 30 testis-enriched genes dispensable for male fertility in mice^†^

**DOI:** 10.1093/biolre/ioz103

**Published:** 2019-06-14

**Authors:** Yonggang Lu, Seiya Oura, Takafumi Matsumura, Asami Oji, Nobuyuki Sakurai, Yoshitaka Fujihara, Keisuke Shimada, Haruhiko Miyata, Tomohiro Tobita, Taichi Noda, Julio M Castaneda, Daiji Kiyozumi, Qian Zhang, Tamara Larasati, Samantha A M Young, Mayo Kodani, Caitlin A Huddleston, Matthew J Robertson, Cristian Coarfa, Ayako Isotani, R John Aitken, Masaru Okabe, Martin M Matzuk, Thomas X Garcia, Masahito Ikawa

**Affiliations:** 1 Department of Experimental Genome Research, Research Institute for Microbial Diseases, Osaka University, Suita, Osaka, Japan; 2 Graduate School of Pharmaceutical Sciences, Osaka University, Suita, Osaka, Japan; 3 RIKEN Center for Biosystems Dynamics Research, Kobe, Hyogo, Japan; 4 Department of Bioscience and Genetics, National Cerebral and Cardiovascular Center, Suita, Osaka, Japan; 5 Graduate School of Medicine, Osaka University, Suita, Osaka, Japan; 6 Immunology Frontier Research Center, Osaka University, Osaka, Japan; 7 Laboratory Animal Center, Chongqing Medical University, Chongqing, China; 8 Priority Research Centre for Reproductive Science, Faculty of Science, The University of Newcastle, Callaghan, New South Wales, Australia; 9 Department of Pathology and Immunology, Baylor College of Medicine, Houston, Texas, USA; 10 Center for Drug Discovery, Baylor College of Medicine, Houston, Texas, USA; 11 Dan L Duncan Comprehensive Cancer Center, Baylor College of Medicine, Houston, Texas, USA; 12 Advanced Technology Cores, Baylor College of Medicine, Houston, Texas, USA; 13 Department of Molecular and Cellular Biology, Baylor College of Medicine, Houston, Texas, USA; 14 Graduate School of Biological Sciences, Nara Institute of Science and Technology, Nara, Japan; 15 Hunter Medical Research Institute, New Lambton Heights, New South Wales, Australia; 16 Department of Molecular and Human Genetics, Baylor College of Medicine, Houston, Texas, USA; 17 Department of Pharmacology and Chemical Biology, Baylor College of Medicine, Houston, Texas, USA; 18 Department of Biology and Biotechnology, University of Houston-Clear Lake, Houston, Texas, USA; 19 The Institute of Medical Science, The University of Tokyo, Tokyo, Japan

**Keywords:** CRISPR/Cas9, knockout mice, male infertility, testis expression, spermatogenesis

## Abstract

More than 1000 genes are predicted to be predominantly expressed in mouse testis, yet many of them remain unstudied in terms of their roles in spermatogenesis and sperm function and their essentiality in male reproduction. Since individually indispensable factors can provide important implications for the diagnosis of genetically related idiopathic male infertility and may serve as candidate targets for the development of nonhormonal male contraceptives, our laboratories continuously analyze the functions of testis-enriched genes in vivo by generating knockout mouse lines using the CRISPR/Cas9 system. The dispensability of genes in male reproduction is easily determined by examining the fecundity of knockout males. During our large-scale screening of essential factors, we knocked out 30 genes that have a strong bias of expression in the testis and are mostly conserved in mammalian species including human. Fertility tests reveal that the mutant males exhibited normal fecundity, suggesting these genes are individually dispensable for male reproduction. Since such functionally redundant genes are of diminished biological and clinical significance, we believe that it is crucial to disseminate this list of genes, along with their phenotypic information, to the scientific community to avoid unnecessary expenditure of time and research funds and duplication of efforts by other laboratories.

## Teaser

Thirty testis-enriched genes are dispensable for male fertility based on phenotypic analyses of knockout mice produced by the CRISPR/Cas9 system.

## Introduction

Globally, one in every six couples is suffering from infertility, with male factors wholly or partly contributing to nearly 50% of overall infertility cases [1, 2]. Male infertility is recognized as a complicated pathological condition that is characterized by an extreme heterogeneous spectrum of phenotypic presentations [3]. Such multifactorial nature of etiology hampers the diagnosis of male infertility and renders the underlying causes of reproductive defects obscure in about half of the cases [4]. According to a recent clinically based etiological categorization, male infertility falls into four major groups: obstruction or dysfunction of the reproductive tract, disturbance of the hypothalamic–pituitary axis, qualitative defects in the semen, and impaired sperm quality and/or quantity [5, 6].

In recent years, genetic disorders emerge as one of the leading causes of male infertility, accounting for at least 15% of male infertility cases and being involved in all four of the major etiologies [5]. Hence, genetic tests such as cytogenetic analysis and mutation detection have been incorporated into routine screening protocols for male infertility [1]. With the development of intracytoplasmic sperm injection (ICSI), natural barriers of insemination (i.e. the processes of sperm migration and penetration through the uterotubal junction, cumulus oophorus, zona pellucida, and oolemma) have been completely bypassed, enabling successful fertilization with defective spermatozoa. However, children conceived by such assisted reproductive technologies may have an elevated risk of congenital anomalies, indicating that the genetic defects underlying male infertility become even more relevant [7]. Thus, the genetic networks underpinning fertility in men should be intensively interrogated for the sake of the reproductive health of male patients and the general well-being of their children [5].

Given the striking similarities between human and mouse genomes, mice have long been a robust tool for the study of human diseases. There are over 2300 genes predominantly expressed in the mouse testis, hundreds of which may facilitate the normal functioning of the male reproductive system or contribute to male infertility [8]. Nevertheless, only a handful of testis-enriched genes so far have been studied in terms of their essentiality for spermatogenesis and/or sperm function in male reproduction. A comprehensive characterization of the molecular networks involved in key reproductive events (e.g. sperm production, sperm motility, capacitation, acrosome reaction, and gamete fusion) would have profound implications in a wide range of context, such as clinical diagnoses of idiopathic male infertility that is of probable genetic origin, rational design of nonhormonal male contraceptives, and fertility control of invasive species.

In absence of a culture system that supports the production of fully functional spermatozoa completely in vitro, the functions of testis-enriched genes can be alternatively reflected in vivo by creating male mice carrying a null mutation in the genes of interest. This approach is particularly feasible to study genes expressed in reproductive organs because in most case, the entire loci can be removed without jeopardizing the viability of the mutant mice [9]. Upon the production of knockout mouse lines, the essentiality of the deleted genes can be readily determined by examining the fertility of their homozygous male offspring. In recent years, by using this in vivo approach, many testis-enriched genes were found to have novel function and to be indispensable for male reproduction [10], such as the intercellular bridge protein TEX14 [11], the sperm cation channel proteins CATSPER [12–14], the oocyte fusion factor IZUMO1 [15], and the component of the sperm flagellar dynein regulatory complex TCTE1 [16]. On the other hand, a number of genes, which had been widely presumed as essential factors for reproduction, turned out to be individually dispensable for fertilization in vivo (e.g. the acrosomal protease Acrosin [17], the zona pellucida binding protein B4GALT1 [18], and the oocyte fusion protein Fertilin [19]; reviewed in [20]). It may be possible that the fertility of knockout mice is derived from compensatory mechanisms supplanting the functions of the targeted genes. However, such functional redundancy renders these candidate genes ineffective as targets for male contraceptive development. Besides, dispensable factors are likely to be clinically irrelevant as patients carrying mutations solely in these genes would not suffer from compromised fertility.

In the era of CRISPR/Cas9-based genome engineering, the generation of genetically modified mice becomes much more flexible, accurate, efficient, and cost-effective. By directly delivering crRNA/tracrRNA/Cas9 ribonucleoprotein complexes into mouse zygotes via electroporation, the timespan required for generating homozygous mutant mice has been greatly shortened and the efficiency of genome editing has been improved. Hence, rapid and mass production of knockout mouse lines becomes feasible, which, in turn, accelerates our progress in identifying key genes underpinning normal male reproductive functions. During our in vivo screening, we discovered 30 testis-enriched and 4 ubiquitously expressed genes that are individually dispensable for male fertility in mice.

## Materials and methods

### Animals

All experiments involving animals were approved by the Institutional Animal Care and Use Committees of Osaka University (Osaka, Japan) and Baylor College of Medicine (Houston, TX) and were conducted in compliance with the guidelines and regulations for animal experimentation of both institutions. In this study, all wild-type B6D2F1/J and ICR mice used in the laboratory of M.I. were purchased from CLEA Japan, Inc. (Tokyo, Japan) or Japan SLC, Inc. (Shizuoka, Japan). All mutant mouse lines were generated in the laboratory of M.I. on the genetic background of B6D2F1/J. The mice used in the laboratory of M.M.M. were produced from intercrosses between C57BL6 and 129S6/SvEv mice to analyze the patterns of tissue expression for all candidate genes.

### Conservation of candidate genes among species

Identification of conserved genes was performed using Ensembl Compara [21], PSI-Blast, tBlastN, and OrthoDB [22] searches. Potential orthologs (e.g. predicted genes from draft genomes) were individually paired with the mouse gene using Align Sequence Protein Blast. Evolutionary rates among mammals were obtained from OrthoDB (release 10).

### Digital PCR

Sequences for different tissues were downloaded from the Sequence Read Archive (SRA), trimmed using TrimGalore, and aligned against the human genome (GRCh38) or mouse genome (GRCm38) using HISAT2. The gene expression in each tissue was quantified using featureCounts, and tissues were batch corrected by removing unwanted variation using RUVR. Differential gene expression was determined for each nonreproductive tissue against each reproductive tissue using EdgeR. The data are comprised 5 human testis datasets [23], 18 purified human germ cell datasets [24], 6 human epididymis segment datasets [25], 9 mouse testis datasets [26, 27], and 6 purified mouse germ cell datasets [27]. An additional 118 datasets contributed to the 26 nonreproductive human tissues [23] and 62 datasets contributed to the 14 nonreproductive mouse tissues [26]. The SRA value for each dataset, the fold changes, and false detection rates are also provided in Supplementary Excel files 1 and 2.

### Generation of knockout mice by CRISPR/Cas9 system

In this study, all knockout mouse lines were generated by the CRISPR/Cas9 genome editing technology. Generally, single-guide RNAs (sgRNAs) were designed using the web tool CRISPRdirect (crispr.dbcls.jp [28]) and their cleavage efficiency was evaluated by transfecting HEK293T cells with pCAG-EGxxFP (Addgene #50716) and pSpCas9(BB)-2A-Puro (pX459) V2.0 (Addgene #62988) plasmids as previously described [29]. Wild-type embryos were genetically modified in vitro through CRISPR/Cas9-mediated modifications of zygotes or ES cells.

For the zygote approach, B6D2F1/J females were hormonally primed and paired with wild-type B6D2F1/J males. Zygotes carrying indel/null mutations in the genes of interest were generated by (1) microinjecting pX459 plasmids expressing sgRNAs and Cas9 into the pronuclei of zygotes [29] or (2) electroporating two-pronuclear zygotes with crRNA/tracrRNA/Cas9 ribonucleoprotein complexes using a NEPA21 Super Electroporator (NEPAGENE, Chiba, Japan) [30]. The treated embryos were cultured in KSOM medium to the two-cell stage and transplanted into the oviducts of pseudopregnant ICR females at 0.5 day after mating with vasectomized males. The mutant F0 mice carrying large deletions or small indels were identified by genomic PCR or a MultiNA Microchip Electrophoresis System (Shimadzu, Kyoto, Japan), respectively. The DNA sequence of mutant alleles was further confirmed by Sanger sequencing. After genotype validation, heterozygous F0 mice underwent serial mating to generate homozygous or compound heterozygous mutant offspring. The knockout mouse lines that were generated by the zygote approach are indicated in Supplementary Table S1.

For the ES cell approach, EGR-G101 ES cell line, which was established previously from C57BL/6-Tg (CAG/Acr-EGFP) C3-N01-FJ002Osb, was seeded on mouse embryonic fibroblasts and transfected with pX459 and pPGKpuro (Addgene #11349) plasmids using Lipofectamine LTX & PLUS reagents (Life Technologies, Carlsbad, CA). Transfected ES cells were transiently selected with puromycin, and the resultant ES cell clones were subject to genotyping with PCR and Sanger sequencing. The positive clones were expanded to analyze their karyotypes. The mutant ES cell clones with normal karyotypes were microinjected into wild-type 8-cell ICR embryos. The chimeric embryos were cultured in vitro until blastocyst stage and transplanted into the uterine horns of pseudopregnant ICR females at 2.5 days after mating with vasectomized males [31, 32]. Chimeric male mice, which were identified by the dark pigmentation in the eyes and coat, were paired with wild-type B6D2F1/J females for germline transmission. In the case of failed germline transmission, ICSI was employed to produce heterozygous mutant offspring. The knockout mouse lines generated with the ES cell approach are listed in Supplementary Table S2.

The primers and PCR conditions used for genotyping the knockout mice generated by the CRISPR/Cas9 system are enumerated in Supplementary Table S3.

### Fertility test for knockout male mice

Upon sexual maturation, F2 homozygous or compound heterozygous knockout male mice were caged individually with two to three 6-week-old wild-type B6D2F1/J female mice for at least 4 weeks. Two to three males were tested for each knockout line to meet the requirements for statistical validity. The fecundity of wild-type B6D2F1/J was tested in parallel as positive controls. After the mating period, male mice were removed from the cages and wild-type females were kept for another 3 weeks to allow them to deliver their final litters. During the fertility test, the number of pups was counted at birth. The average litter size for each mouse line was calculated by dividing the total number of pups with the number of litters.

### Testicular and sperm analyses for knockout male mice

After the fertility test, three knockout male mice and three same-aged wild-type B6D2F1/J mice were euthanized by cervical dislocation following anesthesia to examine their testis weights, testicular and epididymal histology, sperm morphology, and sperm motility. Testes and epididymides were fixed in Bouin fluid (Polysciences, Inc., Warrington, PA), embedded in paraffin wax, sectioned at a thickness of 5 μm on a Microm HM325 microtome (Microm, Walldorf, Germany), and stained with 1% periodic acid (Nacalai Tesque, Kyoto, Japan) and Schiff's reagent (Wako, Osaka, Japan) followed by counterstaining with Mayer's hematoxylin solution (Wako, Osaka, Japan). Spermatozoa were extracted from cauda epididymis and dispersed in TYH medium for 10 min. Sperm morphology was observed under an Olympus BX53 differential interference contrast microscope equipped with an Olympus DP74 color camera (Olympus, Tokyo, Japan). Sperm motility was measured with the CEROS II sperm analysis system (Hamilton Thorne Biosciences, Beverly, MA) at 10 min and 2 h of incubation.

### Statistical analysis

The statistical difference was determined using the Student t-test. Differences were considered statistically significant if the *P* value was less than 0.05. All statistical analyses were conducted using Microsoft Office Excel (Microsoft Corporation, Redmond, WA).

## Results

### In silico screening of candidate genes for in vivo functional analysis

Similar to the bioinformatics analyses described previously [9], genes that may have predominant or specific expression in testis were screened in silico according to multiple public databases. For example, the expressed sequence tag (EST) profile in NCBI's UniGene database (ncbi.nlm.nih.gov/unigene) and the RNA profiling data generated by the Mouse ENCODE project (ncbi.nlm.nih.gov/gene) gives the TPM (transcripts per million) and RPKM (reads per kilobase million) values of expression levels in various tissues and organs, respectively. A heat map showing the predicted expression patterns of all genes was generated based on the TPM values provided by the EST profile (see Supplementary Figure S1). Through these bioinformatic predictions, we identified 30 testis-enriched genes and 4 ubiquitously expressed genes (see Supplementary Table S4) to be analyzed in this study.

In some cases, inconsistent expression patterns of candidate genes were discovered from the databases, rendering the testis-enriched expression pattern inconclusive. Such discrepancies are mainly ascribed to different tissues and organs were analyzed in the two sources. For example, the transcript level of *Fndc8* in testis is lower than in joint according to the EST profile. However, the expression of *Fndc8* in joint has not been analyzed by the Mouse ENCODE project, resulting in a predominant TPM count in testis. In addition, no mouse ENCODE transcriptome data was available for *Hmgb4, Oxct2a, Oxct2b, Scp2d1, Tgif2lx1, Trpd52l3, Ube2d2b*, and *Ubqln5* (Supplementary Table S4).

**Figure 1.**
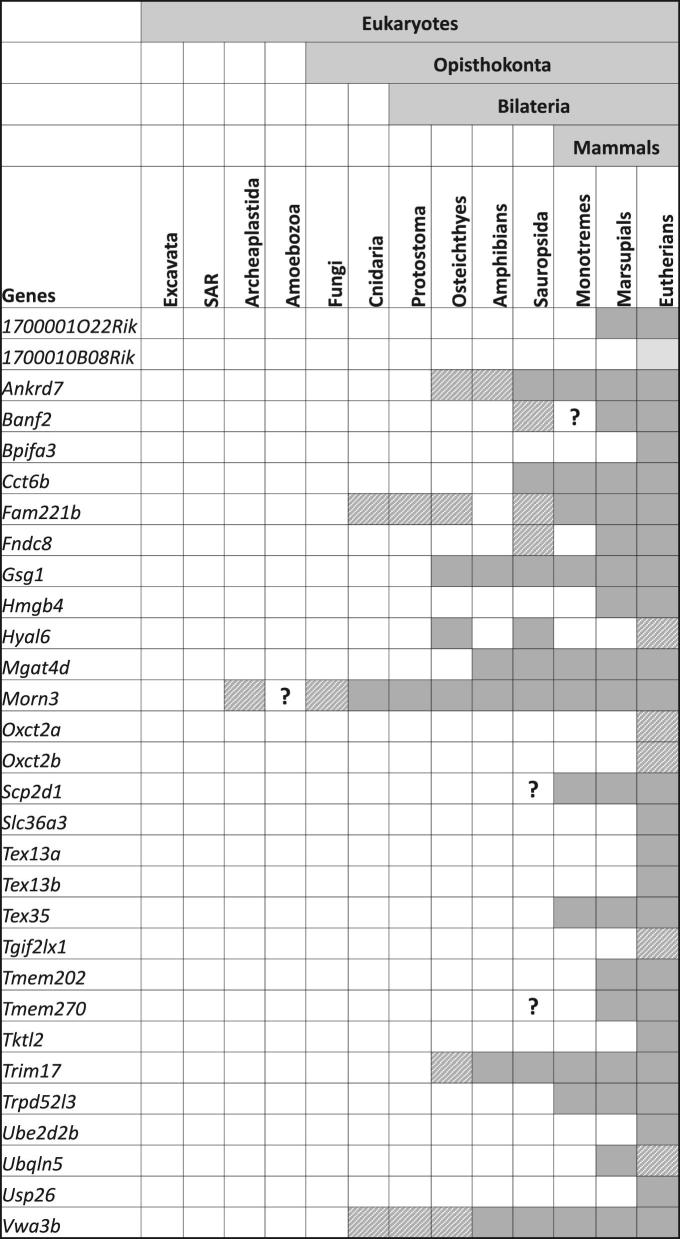
Conservation of the testis-enriched genes among species. The presence of orthologs is indicated by solid or shaded squares. Orthologous genes conserved in all species in a taxon are highlighted with solid dark gray. Shaded gray indicates loss of an ortholog in several species within a taxon. Solid light gray indicates that *1700010B08Rik* is only found in mice. Question marks indicate potential orthologs in species within a taxon.

The primary focus was given to genes that have conserved open reading frames (ORFs) in both human and mice. According to the NCBI search, the majority of the 30 testis-enriched mouse genes possess human orthologs, except for *1700010B08Rik, Hyal6, Ube2d2b*, and *Ubqln5* (Supplementary Table S5). Different from human *OXCT2*, the mouse has two paralogs, *Oxct2a* and *Oxct2b*, that are highly homologous between their overlapping sequences. To understand the extent of evolutionary conservation of the genes of interest, we conducted in silico analysis based on the information collected from multiple databases. Nearly all of the 30 testis-enriched genes are conserved in mammals, particularly in eutherians and marsupials (Figure 1). Exceptionally, *1700010B08Rik*, which is highlighted in yellow, was found to be a mouse-specific gene. *Hyal6* was annotated as a pseudogene in human. *Ubqln5* presents in all eutherians and marsupials except for simians, including human. The conservation of the 4 ubiquitously expressed genes is presented in Supplementary Figure S2. The evolutionary rates of all genes were summarized as a heat map based on the OrthoDB database (Supplementary Figure S3).

### Confirmation of expression patterns using digital PCR

**Figure 2.**
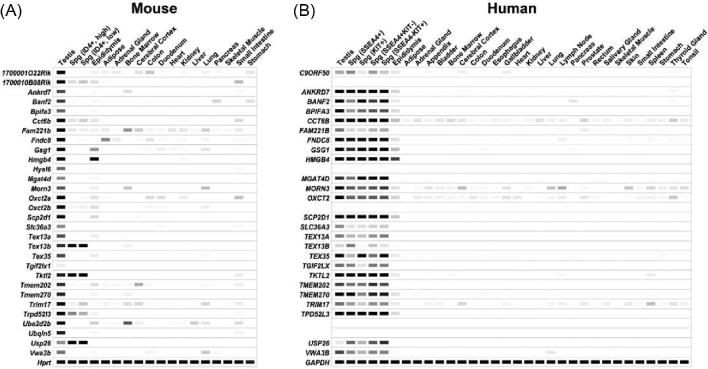
Digital PCR depicting the average TPM value per tissue per gene from 240 published mouse and human RNA-seq datasets. (A) Patterns of gene expression in mouse tissues and organs. (B) Patterns of gene expression in human tissues and organs. White = 0 TPM, black ≥ 30 TPM.

Given the occasional discrepancies regarding the expression levels provided by the various databases, we performed digital PCR to validate the expression patterns of all candidate genes. As shown in Figure 2, all 30 genes showed predominant or restricted expression in both mouse and human testes. Since the threshold of elevated expression was set to 30 (i.e. TPM values higher than the threshold were presented as darker bands), some genes seemed to have a bias of expression toward other tissues or organs apart from testis. In fact, while *Ube2d2b* showed a high TPM of 208.58 in testis, its transcript level in bone marrow was only 22.49. Additionally, *Tex13b, Tktl2*, and *Usp26* showed an eminent expression in spermatogonia of both mouse and human. The analyses of the expression patterns of the 4 ubiquitously expressed genes are shown in Supplementary Figure S4.

### In vivo functional analyses of candidate genes

To investigate the essentiality of all candidate genes in male reproduction, the majority of the 34 candidate genes, including the 4 ubiquitously expressed ones, were deleted individually by CRISPR/Cas9-mediated zygote or ES cell approaches. Exceptions to this, the two paralogs of human *OXCT2, Oxct2a* and *Oxct2b*, were knocked out simultaneously given that both their ORFs and flanking regions are highly conserved. In this study, homozygous or compound heterozygous mutant mice were obtained from sequential mating and validated by genomic PCR and Sanger sequencing. The outcomes of Sanger sequencing for the mutated loci are summarized in Supplementary Table S6. An example is presented in Supplementary Figure S7 for the Sanger sequencing of *Hmgb4* wild-type, heterozygous, and homozygous mice. While the entire loci were mostly deleted in some knockout lines (e.g. *Gsg1, Slc36a3*, and *Usp26*) with two sgRNAs, other genes (e.g. *Fndc8* and *Hmgb4*) were disabled by introducing frameshift indels using one sgRNA. Notably, *Mgat4d* knockout mouse line carried a 36 bp deletion. In fact, 32 bp was removed in the first exon of *Mgat4d* and the other 4 bp deletion was in its immediate upstream intron. Thus, an inframe deletion that may result in the production of truncated protein was not introduced.

**Figure 3.**
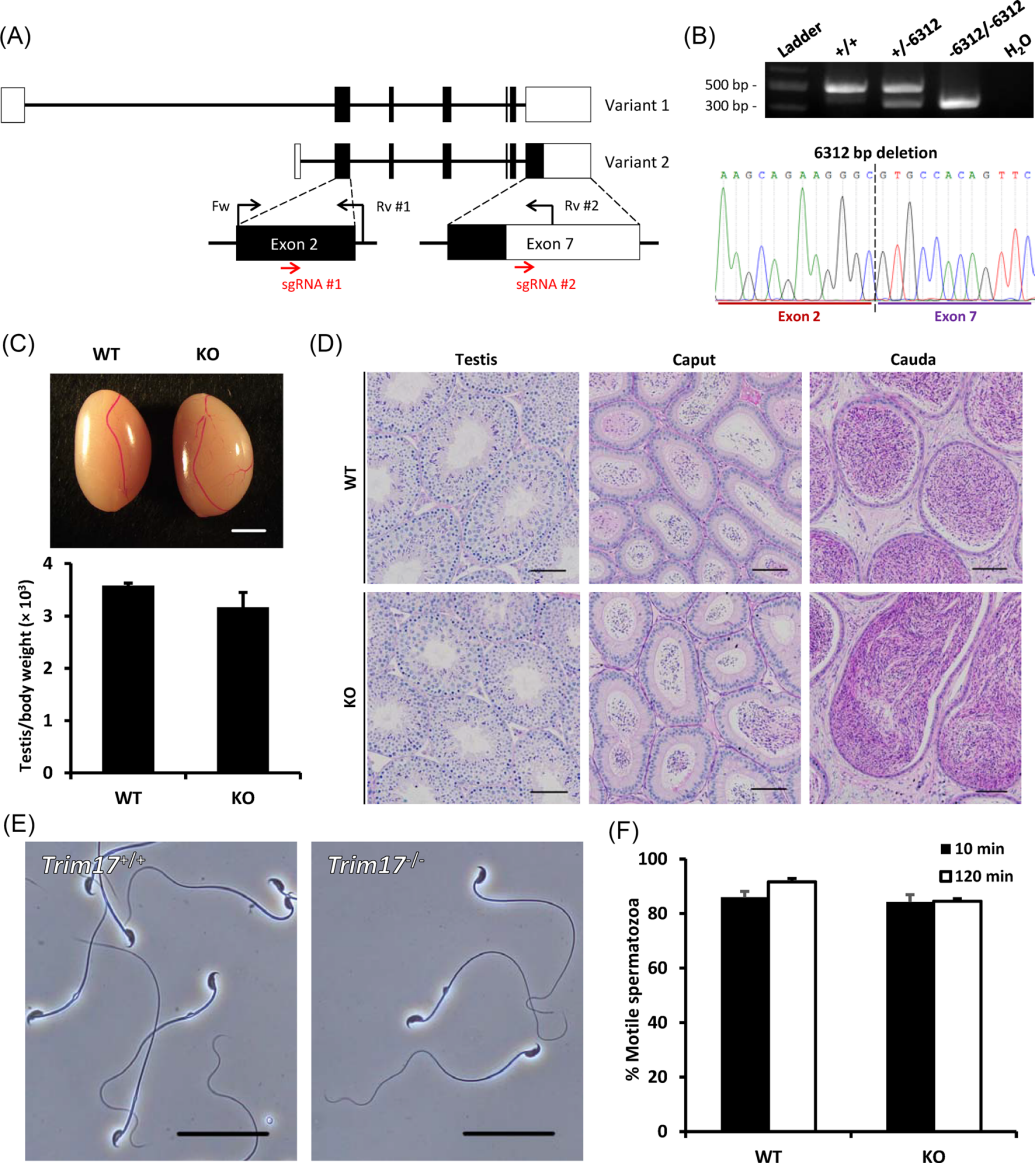
Phenotypic analysis of *Trim17* knockout male mice. (A) Genomic structure and knockout strategy of mouse *Trim17*. Dual sgRNAs were designed to target the first coding exon and the 3^΄^ UTR region downstream the last exon. Fw, forward primer for genotyping; Rv #1/#2, reverse primers for genotyping. (B) Genotype validation of *Trim17* knockout mice by genomic PCR and Sanger sequencing. (C) Comparison of testis size and testis to body weight ratios of *Trim17* wild-type (WT) and knockout (KO) mice. Scale bar = 2 mm. (D) Histological analyses of testes and epididymides in *Trim17* wild-type and knockout mice. Scale bars = 50 μm. (E) Morphology of spermatozoa extracted from the cauda epididymides in *Trim17* wild-type and knockout mice. Scale bars = 100 μm. (F) Percentages of motile spermatozoa in *Trim17* wild-type and knockout mice. Sperm motility was measured at 10 and 120 min of incubation in TYH media.

**Figure 4.**
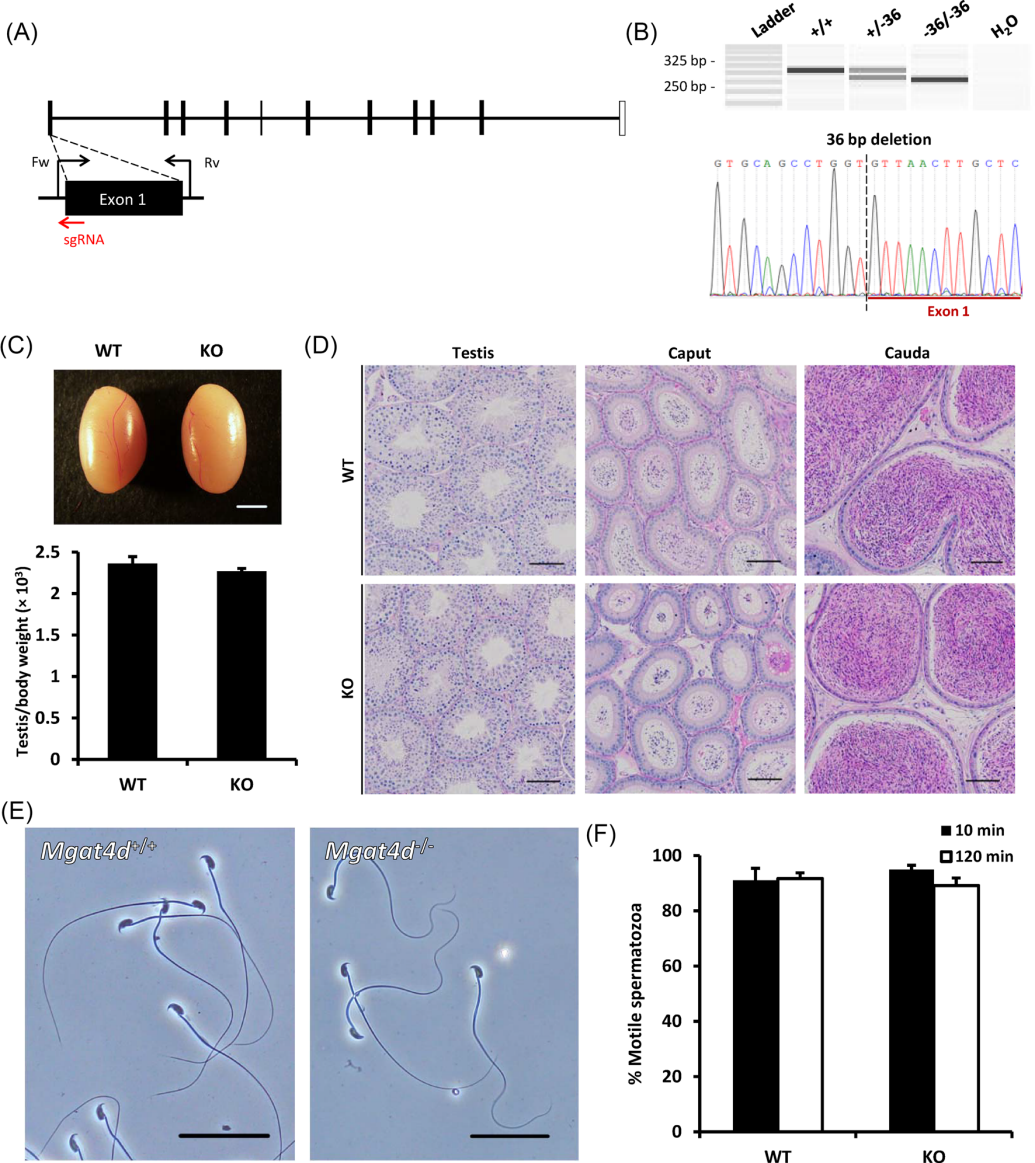
Phenotypic analysis of *Mgat4d* knockout male mice. (A) Genomic structure and knockout strategy of mouse *Mgat4d*. Fw, forward primer for genotyping; Rv, reverse primers for genotyping. (B) Genotype validation of *Mgat4d* knockout mice by MultiNA microchip electrophoresis and Sanger sequencing. (C) Comparison of testis size and testis to body weight ratios of *Mgat4d* wild-type (WT) and knockout (KO) mice. Scale bar = 2 mm. (D) Histological analyses of testes and epididymides in *Mgat4d* wild-type and knockout mice. Scale bars = 100 μm. (E) Morphology of spermatozoa extracted from the cauda epididymides in *Mgat4d* wild-type and knockout mice. Scale bars = 50 μm. (F) Percentages of motile spermatozoa in *Mgat4d* wild-type and knockout mice. Sperm motility was measured at 10 and 120 min of incubation in TYH media.

Phenotypic analyses were carried out for the knockout male mice in parallel with same-aged wild-type controls to investigate the development of the testicles and spermatozoa in absence of the genes of interest. No abnormal development or behavior was observed in any of the knockout mouse lines generated in this study. Herein, *Trim17* and *Mgat4d* knockout lines were used as examples for in vivo functional analyses of knockout male mice generated by the CRISPR/Cas9 system (Figures 3 and 4).

There are two transcriptional variants of *Trim17* according to the Ensembl genome browser. To knockout *Trim17* completely, sgRNAs were designed to target the first coding exon (exon 2) of both variants and the 3’ UTR region downstream the coding region of exon 7 in variant 2, respectively (Figure 3A). Two sgRNAs targeting the two exons were selected based on the outcomes of EGxxFP reporter assay and off-target prediction using CRISPRdirect. Through in vitro electroporation, the crRNA/tracrRNA/Cas9 ribonucleoprotein complex was introduced into two pronuclear zygotes, which were subsequently transferred to the oviducts of pseudopregnant ICR females. Using the three primers presented in Figure 3A and Supplementary Table S3, mutant offspring carrying the knockout allele were identified with genomic PCR and Sanger sequencing (Figure 3B). No significant difference was observed between the wild-type and knockout testes in their sizes and weights (Figure 3C). Histological examination of testes and epididymides in wild-type and knockout mice was conducted to investigate potential pathological abnormalities derived from the mutation in the genome. However, no abnormality was observed in terms of the composition, quantity, and morphology of spermatogenic cells in the seminiferous tubules at each stage and spermatozoa in the caput and cauda epididymides (Figure 3D). By observing the extracts of wild-type and knockout cauda epididymides under a light microscope, we further confirmed that the depletion of *Trim17* did not affect the morphology of mature spermatozoa (Figure 3E). Computer-assisted sperm analysis (CASA) revealed that the motility of *Trim17*-null spermatozoa was comparable to that of wild-type spermatozoa (Figure 3F).

To disrupt *Mgat4d*, a sgRNA was employed to introduce a frameshift mutation in its first coding exon (exon 1; Figure 4A). The cleavage efficiency of the candidate sgRNAs was evaluated in vitro through the EGxxFP reporter assay. The CRISPR/Cas9 component was introduced into zygotes by microinjecting pX459 plasmid encoding the sgRNA and Cas9 into the pronuclei. Using the primers indicated in Figure 4A and Supplementary Table S3, mutant offspring carrying the 36 bp deletion were detected using the MultiNA microchip electrophoresis system (Figure 4B). Phenotypic analyses revealed that *Mgat4d* knockout mice exhibited normal testicular size and weight (Figure 4C), normal spermatogenesis and testicular and epididymal histology (Figure 3D), and normal sperm morphology and motility (Figure 4E and F).

### Fertility tests for knockout male mice

To examine the fertility of the adult male mice lacking the genes of interest, they were caged with two to three wild-type females for at least 1 month. The average litter sizes revealed that all knockout males were able to sire normal numbers of pups during the mating period and there was no statistically significant difference between the fecundity of knockout and wild-type males (Table 1 and Supplementary Table S7). The mouse lines knocked out in this study have been deposited as frozen sperm to the RIKEN BioResource Research Center and Center for Animal Resources and Development at Kumatomo University, where they will be made available to all researchers (Supplementary Table S4).

**Table 1.** Outcomes of the fertility tests for the 29 knockout mouse lines.

Gene symbol	Genotype	No. of males	No. of pups	No. of litters	Mating period	Average litter size ± SD
Wild type	+/+	3	214	21	10 weeks	10.2 ± 2.0
*1700001O22Rik*	+1/–1	2	119	12	10 weeks	9.9 ± 1.3
*1700010B08Rik*	–3+19/–3+19	3	144	15	10 weeks	9.6 ± 2.0
*Ankrd7*	–8/–8	2	135	16	11 weeks	8.4 ± 2.7
*Banf2*	–17/–17	4	126	13	18 weeks	9.7 ± 1.5
*Bpifa3*	–8110/–8110	3	132	15	11 weeks	8.8 ± 1.3
*Cct6b*	–44827/–44827	3	102	11	6 weeks	8.5 ± 1.0
*Fam221b*	–6315/–6314+1	3	184	18	9 weeks	9.0 ± 1.9
	–6315/–6312					
*Fndc8*	–1/–1	3	150	16	15 weeks	9.4 ± 1.7
*Gsg1*	–4636/–4636	3	156	20	11 weeks	7.8 ± 2.9
*Hmgb4*	–7/–7	3	136	13	8 weeks	10.5 ± 1.3
*Hyal6*	–17/–17	2	122	11	6 weeks	11.1 ± 0.9
*Mgat4d*	–36/–36	3	186	22	9 weeks	8.6 ± 1.9
*Morn3*	–2+1/–2+1	2	118	13	8 weeks	9.1 ± 2.3
*Oxct2a; Oxct2b*	[–1/–1]; [–1722/–1722]	3	251	27	8 weeks	9.3 ± 2.3
*Scp2d1*	–594/–594	3	103	10	7 weeks	10.3 ± 1.1
*Slc36a3*	–26893/–26893	3	110	16	11 weeks	8.0 ± 2.1
*Tex13a*	–1861/Y	3	139	14	11 weeks	9.9 ± 1.1
*Tex13b*	–3340/Y	2	80	10	9 weeks	8.0 ± 2.6
*Tex35*	–9421/–9421	3	110	16	11 weeks	7.4 ± 2.5
*Tgif2lx1*	–80/Y	3	51	7	11 weeks	7.3 ± 2.0
*Tktl2*	–1748/–1748	3	176	21	9 weeks	8.4 ± 2.2
*Tmem202*	‐6002/‐6006	3	161	16	8 weeks	8.5 ± 1.4
	–6002/–6003+7					
*Tmem270*	–4985/–4985	3	139	14	7 weeks	9.9 ± 1.3
*Trim17*	–6312/–6312	3	192	20	9 weeks	9.6 ± 3.3
*Trpd52l3*	–749/–749	2	154	18	11 weeks	8.6 ± 0.6
*Ube2d2b*	–397/–397	3	86	8	6 weeks	10.8 ± 1.4
*Ubqln5*	–8/–8	2	87	9	11 weeks	9.7 ± 2.8
*Usp26*	–1/Y	3	80	10	15 weeks	8.0 ± 3.6
*Vwa3b*	–17/–17	2	102	13	8 weeks	7.8 ± 2.8

*Oxct2a* and *Oxct2b* double knockout line has 1 bp deletion in *Oxct2a* and 1722 bp deletion in *Oxct2b*. Compound heterozygotes carrying different mutations were used for testing the fertility of *Fam221b* (–6315/–6314+1 and –6315/–6312) and *Tmem202* (–6002/–6006 and –6002/–6003+7) knockout males.

## Discussion

There are many public databases available for screening genes that have an expression bias toward testis. From the Human Protein Atlas, 1079 genes have been defined as testis-enriched genes and 2237 genes are recognized to have a higher expression in the testis compared with other tissues and organs (proteinatlas.org/humanproteome/tissue/testis; updated on May 8, 2019). From the UniGene database, by searching random known testis-enriched genes and selecting “Show more entries with profiles like this,” 1070 mouse genes that are predominantly expressed in testis are identified (updated on May 8, 2019).

Additional information on the proteins encoded by the genes of interest was gathered for predicting the gene function and potential involvement in reproduction. The “Protein Information” section in the Mouse Genome Informatics database (MGI; informatics.jax.org), UniProt (uniprot.org), Simple Modular Architecture Research Tool (SMART; smart.embl-heidelberg.de), and NCBI's Protein database (ncbi.nlm.nih.gov/protein) provide substantial information on the protein features, such as protein family, conserved domains, posttranslational modification, interacting proteins, and subcellular localization. The web tools TMHMM2.0 (cbs.dtu.dk/services/TMHMM [33]), SignalP5.0 (cbs.dtu.dk/services/SignalP [34]) and SOSUI (harrier.nagahama-i-bio.ac.jp/sosui [35]) also facilitate predicting the number of transmembrane domains, the presence of a signal peptide, and the protein's secondary structure, respectively. Among the 34 genes analyzed in this study, 26 genes were predicted to encode soluble proteins that have no transmembrane domain and the remaining 7 genes encode proteins with one or more transmembrane helices (see Supplementary Table S5). *Bpifa3* and *Oxct2b* were predicted to encode signal peptides but no transmembrane domain, implying that they are secreted proteins synthesized in the endoplasmic reticulum and transported to the extracellular space. The single-pass transmembrane protein HYAL6 was also predicted to have a signal peptide, suggesting this protein localizes to the membrane as a type I transmembrane protein.

In this study, all knockout mouse lines were maintained on the genetic background of B6D2F1/J and generated using the CRISPR/Cas9 genome engineering system. Since a same mutation may exhibit phenotype with varying degrees of severity in mice raised on different types of pure backgrounds, the use of a mixed background would increase the screening stringency for identifying genes that are individually indispensable for male fertility and are promising targets for male contraception. Microinjection of circular plasmids encoding sgRNA and Cas9 into the pronuclei of mouse zygotes was previously used in the laboratory of M.I. to produce mutant mouse lines. This zygote approach, which now utilizes in vitro electroporation, greatly simplifies the procedures and shortens the time required for generating knockout mice. We successfully knocked out genes that are up to 240 kb but failed to delete larger targets over 300 kb using the in vitro electroporation approach (unpublished data). Thus, the ES cell approach is of immense importance in securing the success of complicated genome editing. To address the concerns of truncated proteins being produced via transcriptional splicing, during our more recent screening, we tend to remove the entire loci of interest using dual sgRNAs instead of introducing frameshift mutations using one sgRNA. In addition, recent reports stress that nonsense-mediated mRNA decay-induced degradation of mutant mRNA transcripts may trigger transcriptional adaptation, where related genes exhibiting sequence similarity are upregulated to compensate the function of the mutated genes [36, 37]. Such genetic compensatory mechanism explains why certain gene knockouts show less severe phenotype than corresponding gene knockdowns. However, it remains unknown whether this compensatory mechanism is a feature that applies to most genes.

A number of the genes knocked out in this study have been reported previously in terms of their subcellular localization and putative roles in male reproduction. *Ankrd7* mRNA was restricted in its expression to mouse testis after day 14 of postnatal development, and ANKRD7 protein is localized to the nuclei of mature Sertoli cells, implying its potential involvement in the maturation of Sertoli cells [38]. However, our functional analysis revealed that the depletion of this gene did not compromise the fecundity of knockout males, suggesting Sertoli cells can develop normally in absence of ANKRD7 in vivo.

As indicated by Choi et al. [39], the expression of *Gsg1* mRNA was detected in the endoplasmic reticulum of mouse testis after day 24 of postnatal development. This protein was found to interact with TPAP, a testis-specific cytoplasmic polyadenylate polymerase indispensable for spermatogenesis, in vitro and to direct the localization of TPAP from the cytosol to ER in cultured cells [39]. *Gsg1* knockout males exhibited normal fecundity, implying that TPAP can function normally in vivo in the absence of GSG1.

HMGB4 had been identified as a testis-specific protein that is initially expressed in mouse testis at 2 weeks postpartum and localizes to the basal pole of the nuclei of elongating spermatids [40]. Such basal localization of HMGB4 disappears in spermatids lacking H1FNT (H1 histone family, member N, testis-specific), a protein localized to the apical pole. The authors hypothesized that HMGB4 and H1FNT may collectively determine the spatial organization of chromatin in elongating spermatids, which is important for the proper condensation of chromatin during spermiogenesis. Moreover, the depletion of *Hmgb2* resulted in abnormal localization of HMGB4 and H1FNT in elongated spermatids. Therefore, the organization and condensation of chromatin in elongation spermatids could be a complex process that is regulated by a network of genes including *Hmgb2, Hmgb4*, and *H1fnt*. It is not surprising that solely knocking out *Hmgb4* would not affect the spermiogenesis in male mice.

*Morn3* was found to be predominantly expressed in mouse testes and localized to the acrosome and the manchette of elongation of the spermatids [41]. The expression of MORN3 in the manchette was significantly decreased in male mice lacking *Meig1* (meiosis expressed gene 1), an essential factor for spermiogenesis [41, 42]. Therefore, the authors hypothesized that MEIG1 controls the localization of MORN3 in the manchette and MORN3 underpins the formation and function of manchette in accompany with MEIG1 [41]. The fertility test in this study revealed that *Morn3* knockout males had normal fecundity, suggesting the lack of *Morn3* will not jeopardize the formation of the manchette during spermiogenesis.

TEX13B (referred to as TEX13 in [43]) was reported to be specifically expressed in the nuclei of spermatogenic cells and has transcriptional repressor activity in its C-terminus. Overexpression of TEX13B in cultured cells alters the expression levels of 130 genes allowing the authors to propose that *Tex13b* may be involved in transcriptional regulation during spermatogenesis. However, our knockout mouse models reveal that *Tex13a* and *Tex13b* are individually dispensable for male fertility. Since there is a considerable similarity in the C-termini of TEX13A and TEX13B, it is tempting to speculate that these two proteins may be functionally redundant. Generation of *Tex13a* and *Tex13b* double knockout mice is necessary to interrogate if these two genes are collectively essential for transcriptional regulation during spermatogenesis.

In this study, we analyzed the function of 30 testis-enriched genes and 4 ubiquitously expressed genes in male reproduction by generating knockout mouse lines using the CRISPR/Cas9 system. The knockout males exhibited normal fecundity suggests that these 34 genes are individually dispensable for male fertility. Although it is undeniable that these genes may be involved in important reproductive events in association with other unknown factors, their redundant nature diminishes their clinical significance and renders them less effective as targets for male contraception. Alternatively, the restricted and elevated expression in certain spermatogenic cell types enables some genes to serve as potential biomarkers for reproductive diseases in men, such as Sertoli cell only syndrome. Overall, our in vivo functional analysis of testis-enriched genes has been demonstrated to be a robust system for the screening of individually indispensable factors for male fertility, where a single loss-of-function mutation would severely jeopardize normal male reproduction.

## Supplementary data

**Supplementary Figure S1.** Heat map showing the expression patterns of all 34 genes in multiple tissues and organs. Expression levels are based on the TPM values provided by NCBI's UniGene database.

**Supplementary Figure S2.** Conservation of the 4 ubiquitously expressed genes among species. The presence of orthologs is indicated by colored or shaded squares. A gene conserved in all species in a taxon is highlighted with solid green. Shaded green indicates loss of ortholog in several species within a taxon. Question mark indicates potential orthologs in species within a taxon. *Tmem19* is presented in all taxa.

**Supplementary Figure S3.** Evolutionary rates of the 30 testis-enriched genes and 4 ubiquitously expressed genes predicted by OrthoDB.

**Supplementary Figure S4.** The expression patterns of the 4 ubiquitously expressed genes in multiple tissues and organs revealed by digital RT-PCR. (A) The patterns of gene expression in mice. (B) The patterns of gene expression in human.

**Supplementary Figure S5.** Representative genotyping of knockout mice that were generated using the CRISPR/Cas9 system. Sanger sequencing of *Hmgb4* (A) wild-type, (B) heterozygous, and (C) homozygous mice. The deleted region is highlighted in red. Heterozygous deletion of *Hmgb4* in one allele results in overlapping waves.

**Supplementary Table S1.** Single-guide RNAs used for generating knockout mice through the zygote approach and efficiency of embryo transplantation and genome editing. When two sgRNAs were used to delete the entire locus, sgRNAs targeting the gene's upstream (U) and downstream (D) regions are presented. Total pups/embryos transplanted are the number of total pups delivered by pseudopregnant recipient mice divided by the number of total embryos used for oviduct transplantation. GM pups/pups genotyped refers to the number of pups carrying enzymatic mutations divided by the number of pups subjected to genotyping.

**Supplementary Table S2.** Single-guide RNAs used for generating knockout mice through the ES cell approach and efficiency of gene editing and chimera formation. Gene targeting efficiency is determined by dividing the number of ES cell clones with mutated alleles with the number of clones picked for screening. The germline transmission (GLT) of the *Ube2d2b* knockout line was achieved with the assistance of ICSI.

**Supplementary Table S3.** Primers and PCR conditions used for genotype validation of CRISPR/Cas9-derived mutant mice.

**Supplementary Table S4.** The 34 mouse genes knocked out in this study. The TPM and RPKM values indicate the gene expression levels in mouse testis. TPM values are provided by EST profile in NCBI's UniGene database, while RPKM values are obtained from RNA profiling data generated by the Mouse ENCODE project. The RBRC No. and CARD ID are available for the mouse lines that have been deposited as frozen sperm to Riken BioResource Research Center and Center for Animal Resources and Development at Kumamoto University, respectively.

**Supplementary Table S5.** Additional information about the 34 mouse genes analyzed in this study. The presence of transmembrane (TM) domains and signal peptide is predicted using TMHMM and SignalP, respectively.

**Supplementary Table S6.** Detailed genotype of CRISPR/Cas9-derived mutant mice determined by Sanger sequencing. Bases in uppercase and lowercase indicate exon and intron sequences, respectively.

**Supplementary Table S7.** Outcomes of fertility tests for the 4 ubiquitously expressed gene knockout mouse lines.

ioz103_Supplement_File

## Notes

Edited by Dr. Kyle Orwig, PhD, University of Pittsburgh
