# Supplementary material for: CRISPR/Cas9-mediated genome editing reveals 30 testis-enriched genes dispensable for male fertility in mice
Source: Biol Reprod. 2019 Jun 14;101(2):501–11. doi: 10.1093/biolre/ioz103 (PMC6735960; doi:10.1093/biolre/ioz103)
Supplement: ioz103_Supplement_File [file ioz103_supplement_file.docx]

**Supplementary Table S1.** Single-guide RNAs used for generating knockout mice through the zygote approach and efficiency of embryo transplantation and genome editing. When two sgRNAs were used to delete the entire locus, sgRNAs targeting the gene’s upstream (U) and downstream (D) regions are presented. Total pups/embryos transplanted are the number of total pups delivered by pseudopregnant recipient mice divided by the number of total embryos used for oviduct transplantation. GM pups/pups genotyped refers to the number of pups carrying enzymatic mutations divided by the number of pups subjected to genotyping.

| **Gene** | **sgRNA sequences** | | **PAM** | **KO strategy** | **Total pups/embryos transplanted** | **GM pups/pups genotyped** |
| --- | --- | --- | --- | --- | --- | --- |
| *1700001O22Rik* | GATGTTCCGGCGCCAGTCCA | | TGG | Zygote injection | 7/59 (12%) | 7/7 (100%) |
| *1700010B08Rik* | CATCATGGCGGGAAACATGA | | TGG | Zygote injection | 4/26 (15%) | 1/4 (25%) |
| *Ankrd7* | TGCGGTTTTGGCCAGGCTAA | | TGG | Zygote injection | 7/34 (21%) | 3/7 (43%) |
| *Banf2* | AGATGGACGACATGTCGCCC | | AGG | Zygote injection | 12/62 (19%) | 6/10 (60%) |
| *Cct6b* | U | GCGATCAAAATCGCTAACCC | TGG | Zygote electroporation | 23/97 (24%) | 2/23 (9%) |
|  | D | GTTGATGAAATTATGCGAGC | TGG |  |  |  |
| *Cwh43* | GTCGCAGTCATGCCAGGCCTG | | TGG | Zygote injection | 11/72 (15%) | 2/11 (18%) |
| *Fam221b* | U | GCCCTTGGTGTCGGGATTCA | GGG | Zygote electroporation | 20/60 (33%) | 5/20 (25%) |
|  | D | TCCTCTGTGTGGCCTGCGAC | CGG |  |  |  |
| *Fndc8* | GCGACGGTATTCTGTAAAGT | | GGG | Zygote injection | 6/26 (23%) | 2/6 (33%) |
| *Gsg1* | U | TGTCACCCCACTCTCCGATT | TGG | Zygote electroporation | 42/154 (27%) | 11/42 (26%) |
|  | D | TGTCCTGTAGCGCCGAGTAG | AGG |  |  |  |
| *Hmgb4* | GTAAGAAGACACATTCACCT | | TGG | Zygote injection | 15/53 (28%) | 3/13 (23%) |
| *Hyal6* | AATCCTTTCGAAACTCAGAG | | TGG | Zygote injection | 16/82 (20%) | 3/6 (17%) |
| *Kdm5d* | GCCCAACTGGGCTCAAACAC | | AGG | Zygote injection | 24/90 (27%) | 8/12 (67%) |
| *Mgat4d* | AACGTTCTTGGCCTTCATCG | | TGG | Zygote injection | 13/62 (21%) | 7/11 (64%) |
| *Morn3* | GTGAACGGCGATCACTACGT | | GGG | Zygote injection | 9/45 (20%) | 1/6 (17%) |
| *Scp2d1* | U | CCTGCTTTGATCTTCGCTTG | AGG | Zygote electroporation | 22/73 (30%) | 1/22 (5%) |
|  | D | GAAATCAATCGAACTTGAAT | AGG |  |  |  |
| *Tex13a* | U | GTCTTTGCACGGGTGTTAGG | TGG | Zygote injection | 18/92 (20%) | 4/18 (22%) |
|  | D | ATGCAAACCGGTTCTCAATG | TGG |  |  |  |
| *Tmem19* | AGTGTCGTCACTATCTGTCA | | TGG | Zygote injection | 12/71 (17%) | 6/12 (50%) |
| *Tmem202* | U | ACCTACATCCGGATGTTCTG | TGG | Zygote electroporation | 11/64 (17%) | 5/11 (45%) |
|  | D | GACAATTATCGAGAAAGGAG | TGG |  |  |  |
| *Tmem270* | U | GCCGCTCCCCGGGTCAGATC | TGG | Zygote electroporation | 19/78 (24%) | 2/19 (11%) |
|  | D | CGGGGGCCCCGAGGTCTCCT | GGG |  |  |  |
| *Trim17* | U | CTTCCCCTGCCCCGAGTGCA | GGG | Zygote electroporation | 30/60 (50%) | 10/27 (37%) |
|  | D | GCTTGAAGCCGGGCAGACAC | TGG |  |  |  |
| *Trpd52l3* | U | TGTGTCTACGTCCTGTCGAG | AGG | Zygote electroporation | 7/72 (10%) | 4/5 (80%) |
|  | D | ACAGGTTTGGCAGCCACGCA | AGG |  |  |  |
| *Ubqln5* | CAGGAAATATGGCACGAGCT | | AGG | Zygote injection | 15/48 (31%) | 9/15 (60%) |
| *Usp26* | U | GACTTGAGCATTTATCAATA | TGG | Zygote injection | 10/67 (15%) | 1/10 (10%) |
|  | D | AAGTCAAATGTAGGTAATTC | TGG |  |  |  |
| *Vwa3b* | GCTACAAGCAGCGGATGGAC | | TGG | Zygote injection | 8/77 (10%) | 1/3 (33%) |

**Supplementary Table S2.** Single-guide RNAs used for generating knockout mice through the ES cell approach and efficiency of gene editing and chimera formation. Gene targeting efficiency is determined by dividing the number of ES cell clones with mutated alleles with the number of clones picked for screening. The germline transmission (GLT) of the *Ube2d2b* knockout line was achieved with the assistance of ICSI.

| **Gene** | **sgRNA Sequences** | | **PAM** | **Gene targeting efficiency** (%) | **No. of injected ES cell clones** | **No. of clones**  **contributed to**  **chimeras** | **GLT** |
| --- | --- | --- | --- | --- | --- | --- | --- |
| *Bpifa3* | U | CAGCTGTAAGCCCATCGATC | TGG | 21/32 (66%) | 4 | 2 | Yes |
|  | D | GACTAGATTCAGCTCTATAA | AGG |  |  |  |  |
| *Nudt10* | U | TCGTAGGTCCGCGTCTGGTT | CGG | 13/32 (41%) | 2 | 1 | Yes |
|  | D | CAGCGAGCGCGAGGACGAGG | CGG |  |  |  |  |
| *Oxct2a/Oxct2b* | U | GGAGAGCAGAGACCCCACGT | GGG | 29/128 (23%) | 2 | 2 | Yes |
|  | D | ACTAACTGGAAAATCAGCCC | TGG |  |  |  |  |
| *Slc36a3* | U | GGAGGAAGGTATCATTAGAC | CGG | 26/32 (81%) | 2 | 1 | Yes |
|  | D | TGTGCCTTGGACCTACAAGC | AGG |  |  |  |  |
| *Tex13b* | U | GACATCCTCGCAATTCATGA | TGG | 2/24 (8%) | 2 | 1 | Yes |
|  | D | AGTCTGGTTCGCTCACTTTC | TGG |  |  |  |  |
| *Tex35* | U | TTACAGGCTTAGGCAAGTGC | AGG | 16/32 (50%) | 2 | 1 | Yes |
|  | D | GGAGCTGGTGGTAGGCTCCA | TGG |  |  |  |  |
| *Tgif2lx1* | U | TGTAGTATTTCATAAAGTCC | TGG | 10/32 (31%) | 2 | 1 | Yes |
|  | D | GACAGTCGGGAGCTTCCGAG | AGG |  |  |  |  |
| *Tktl2* | U | TGAGTGGATTCGCAGGCGAT | TGG | 4/24 (17%) | 2 | 1 | Yes |
|  | D | CGGGAAACCTAGCGAGCTAC | TGG |  |  |  |  |
| *Ube2d2b* | U | CCACAAGGAACTGAACGACC | TGG | 3/24 (13%) | 2 | 2 | No |
|  | D | ACAACAGAACAGCTCGGGAA | TGG |  |  |  |  |

**Supplementary Table S3.** Primers and PCR conditions used for genotype validation of CRISPR/Cas9-derived mutant mice.

| **Gene** | **Allele** | **Forward primer** | **Reverse primer** | **Annealing** | **Elongation** | **Band size** |
| --- | --- | --- | --- | --- | --- | --- |
| *1700001O22Rik* | WT | CTTCCCGGCGCTATGGACTG | GAGTCAGGGTTGTCGCGG | 65°C, 30 s | 72°C, 30 s | 434 bp |
|  | KO | CTTCCCGGCGCTATGGACTG | GAGTCAGGGTTGTCGCGG | 65°C, 30 s | 72°C, 30 s | 433 or 435 bp |
| *1700010B08Rik* | WT | TCTCATAGGCTCATAGGGAGTGG | GTAGGCTTTCCAGTAAGACCTTCC | 65°C, 30 s | 72°C, 30 s | 466 bp |
|  | KO | TCTCATAGGCTCATAGGGAGTGG | GTAGGCTTTCCAGTAAGACCTTCC | 65°C, 30 s | 72°C, 30 s | 482 bp |
| *Ankrd7* | WT | CAATTGTGAACAAGGTGGTT | GCCCATTCACATTGTATTTC | 65°C, 30 s | 72°C, 30 s | 311 bp |
|  | KO | CAATTGTGAACAAGGTGGTT | GCCCATTCACATTGTATTTC | 65°C, 30 s | 72°C, 30 s | 303 bp |
| *Banf2* | WT | AAAGTCCCCAGTCGATGCTG | CAACGCACCTTGTTGAAACC | 65°C, 30 s | 72°C, 30 s | 281 bp |
|  | KO | AAAGTCCCCAGTCGATGCTG | CAACGCACCTTGTTGAAACC | 65°C, 30 s | 72°C, 30 s | 264 bp |
| *Bpifa3* | WT | TTCTGGACCGTCTGAACGTC | CCACTCTTTACGCAGAGCCG | 60°C, 30 s | 72°C, 30 s | 518 bp |
|  | KO | AGTTCTATTCCAATTGGACC | TTTGACAGTTCGTGGTGAAC | 60°C, 30 s | 72°C, 30 s | 565 bp |
| *Cct6b* | WT | AGTCCGAGTAGAGGTGGAGGA | TTGACGGAGTCTTTTCTTCG | 65°C, 30 s | 72°C, 30 s | 486 bp |
|  | KO | AGTCCGAGTAGAGGTGGAGGA | CAGGCATCACGACACAATTT | 65°C, 30 s | 72°C, 30 s | 417 bp |
| *Cwh43* | WT | GGGCTGGGTGGCAGGTTTGG | CCCTGGGCGCTCACCTACAG | 65°C, 30 s | 72°C, 30 s | 633 bp |
|  | KO | GGGCTGGGTGGCAGGTTTGG | CCCTGGGCGCTCACCTACAG | 65°C, 30 s | 72°C, 30 s | 626 bp |
| *Fam221b* | WT | TGCCCCGACTTATAGACCCT | GGCATGGGATTCGGGATAGG | 65°C, 30 s | 72°C, 30 s | 425 bp |
|  | KO | TGCCCCGACTTATAGACCCT | CTGTCATAGCCAAACTCCTCCA | 65°C, 30 s | 72°C, 30 s | 445/447 bp or 445/448 bp |
| *Fndc8* | WT | GATTGTGAAGTCAGAGCACTGC | CCTCTCAAAACTGATGTGGGGC | 65°C, 30 s | 72°C, 30 s | 472 bp |
|  | KO | GATTGTGAAGTCAGAGCACTGC | CCTCTCAAAACTGATGTGGGGC | 65°C, 30 s | 72°C, 30 s | 473 bp |
| *Gsg1* | WT | TTCACAGTAGCATGGCAGAAG | GGGGTTGCTTTCTGATTTGT | 65°C, 30 s | 72°C, 30 s | 617 bp |
|  | KO | CTTGGGAATGAATGGTGGTGT | AGGCACAGGGAGGTTCTCTT | 65°C, 30 s | 72°C, 30 s | 481 bp |
| *Hmgb4* | WT | AATTTCACCATAAGTAGGGC | CCAGCCTTCCCAAGACTGGAC | 65°C, 30 s | 72°C, 30 s | 767 bp |
|  | KO | AATTTCACCATAAGTAGGGC | CCAGCCTTCCCAAGACTGGAC | 65°C, 30 s | 72°C, 30 s | 760 bp |
| *Hyal6* | WT | CTCTGAAACCCGCAATGCCTCCTGTGATCA | TGACAGCATCTGCAATGTCGCCGGCACTTT | 65°C, 30 s | 68°C, 20 s | 300 bp |
|  | KO | CTCTGAAACCCGCAATGCCTCCTGTGATCA | TGACAGCATCTGCAATGTCGCCGGCACTTT | 65°C, 30 s | 68°C, 20 s | 283 bp |
| *Kdm5d* | WT | GTGAGTAGGCAATTCCATTCGCCCT | AGCACATTTCTTACCTCCAGTTCATTTAGCC | 65°C, 30 s | 72°C, 30 s | 742 bp |
|  | KO | GTGAGTAGGCAATTCCATTCGCCCT | AGCACATTTCTTACCTCCAGTTCATTTAGCC | 65°C, 30 s | 72°C, 30 s | 728 bp |
| *Mgat4d* | WT | CAGTTTTTCACAGCCTTGCCA | TGGTTTGGTTCATCCTGGAGA | 65°C, 30 s | 72°C, 30 s | 281 bp |
|  | KO | CAGTTTTTCACAGCCTTGCCA | TGGTTTGGTTCATCCTGGAGA | 65°C, 30 s | 72°C, 30 s | 245 bp |
| *Morn3* | WT | AGATGGACCCTAGGAGAATGCAAG | CCCGCTTAGCTTACCGTGTTTC | 65°C, 30 s | 72°C, 30 s | 400 bp |
|  | KO | AGATGGACCCTAGGAGAATGCAAG | CCCGCTTAGCTTACCGTGTTTC | 65°C, 30 s | 72°C, 30 s | 401 bp |
| *Nudt10* | WT | CTCGCTAGGCAGCTGCAAG | CCCAGCTTCAGTTTCTCCAGG | 65°C, 30 s | 72°C, 30 s | 494 bp |
|  | KO | CTCGCTAGGCAGCTGCAAG | CCCAGCTTCAGTTTCTCCAGG | 65°C, 30 s | 72°C, 30 s | 388 bp |
| *Oxct2a* | WT | TCCCTGGCAGTATCCTCCTC | CCAGGTAGAGCTTCTCGCAG | 65°C, 30 s | 72°C, 30 s | 505 bp |
|  | KO | TCCCTGGCAGTATCCTCCTC | CCAGGTAGAGCTTCTCGCAG | 65°C, 30 s | 72°C, 30 s | 504 bp |
| *Oxct2b* | WT | CGGTGCTCACTGCCATTTGT | GTTTCGTGGCTGCAGTGAAG | 65°C, 30 s | 72°C, 2 min | 2165 bp |
|  | KO | CGGTGCTCACTGCCATTTGT | GTTTCGTGGCTGCAGTGAAG | 65°C, 30 s | 72°C, 2 min | 442 bp |
| *Scp2d1* | WT | AGGATTTGTTTGGCCAATGA | TCTATTTGCTGGGTTCCACA | 65°C, 30 s | 72°C, 30 s | 1030 bp |
|  | KO | AGGATTTGTTTGGCCAATGA | TCTATTTGCTGGGTTCCACA | 65°C, 30 s | 72°C, 30 s | 436 bp |
| *Slc36a3* | WT | CCTTGGCTGAGGACGCACTC | TATATCTTGGTGTACTAAGG | 60°C, 30 s | 72°C, 30 s | 508 bp |
|  | KO | GTCCACACAGTCTTCCTGCG | AGGCTGAGGTTGATATCAGC | 68°C, 30 s | 68°C, 30 s | 565 bp |
| *Tex13a* | WT | TGGAAGTTGGAACAGTCGTT | ATGGCTATGATCCAGGACAC | 68°C, 1 min 30 s | 68°C, 1 min 30 s | 2273 bp |
|  | KO | TGGAAGTTGGAACAGTCGTT | ATGGCTATGATCCAGGACAC | 68°C, 1 min 30 s | 68°C, 1 min 30 s | 412 bp |
| *Tex13b* | WT | TTGGTTTCAGGACTGCTCATCTC | GAGATAAGCAAGCCCAAAGAGTG | 65°C, 30 s | 72°C, 2 min | 3935 bp |
|  | KO | TTGGTTTCAGGACTGCTCATCTC | GAGATAAGCAAGCCCAAAGAGTG | 65°C, 30 s | 72°C, 2 min | 595 bp |
| *Tex35* | WT | CTAGGCTTAAGGCCTTTTGG | AGGAGCGGAAGAGGATTTGG | 68°C, 30 s | 68°C, 30 s | 479 bp |
|  | KO | CTAGGCTTAAGGCCTTTTGG | TTTCCCTCCTCGCCAAGGCC | 68°C, 30 s | 68°C, 30 s | 447 bp |
| *Tgif2lx1* | WT | GCAGTGAAAGTCTTCCCTGACTC | CCTCGGATGGATTGGAGTG | 65°C, 30 s | 72°C, 30 s | 472 bp |
|  | KO | GCAGTGAAAGTCTTCCCTGACTC | CCTCGGATGGATTGGAGTG | 65°C, 30 s | 72°C, 30 s | 392 bp |
| *Tktl2* | WT | GTTTGAACTTCTGCCTCAAG | CCCTTGGAGAGGACAAAGCG | 65°C, 45 s | 72°C, 30 s | 508 bp |
|  | KO | GTTTGAACTTCTGCCTCAAG | TTTGACAACGACGTACATTG | 65°C, 45 s | 72°C, 30 s | 764 bp |
| *Tmem19* | WT | GACTTGGTTCATCTGAAATGCT | AGGTTTCGGCAGTGAACA | 65°C, 30 s | 72°C, 30 s | 309 bp |
|  | KO | GACTTGGTTCATCTGAAATGCT | AGGTTTCGGCAGTGAACA | 65°C, 30 s | 72°C, 30 s | 280 bp |
| *Tmem202* | WT | GCCAGTGCCCTTAACCTCTG | AAGAAAGTGGGGCCTGTGAC | 65°C, 30 s | 72°C, 30 s | 459 bp |
|  | KO | GCCAGTGCCCTTAACCTCTG | ATTTATGTGTGCACGCTCGC | 65°C, 30 s | 72°C, 30 s | 419/415 bp or 419/425 bp |
| *Tmem270* | WT | GGCTCTGAGTTTTTTCCCGAT | CACACACACATACACACGGAA | 65°C, 30 s | 72°C, 30 s | 484 bp |
|  | KO | GGCTCTGAGTTTTTTCCCGAT | TTTCTGCATCAGCTTCCAGGT | 65°C, 30 s | 72°C, 30 s | 613 bp |
| *Trim17* | WT | ATGGATGCGGTGGAGCTT | TTGCCTGAACTTCCACTCCC | 65°C, 30 s | 72°C, 30 s | 484 bp |
|  | KO | ATGGATGCGGTGGAGCTT | TCTTGGGGAAGGGTGTTAGAA | 65°C, 30 s | 72°C, 30 s | 404 bp |
| *Trpd52l3* | WT | GTAACCTCTTACGGATCTCC | GGTGTCCTCGTTATCACAAC | 60°C, 30 s | 72°C, 1 min | 1213 bp |
|  | KO | GTAACCTCTTACGGATCTCC | GGTGTCCTCGTTATCACAAC | 60°C, 30 s | 72°C, 1 min | 464 bp |
| *Ube2d2b* | WT | TGATGCGACAACCGATGTGA | GGAAACAGTCCGGTGGGAAT | 65°C, 30 s | 72°C, 30 s | 739 bp |
|  | KO | TGATGCGACAACCGATGTGA | GGAAACAGTCCGGTGGGAAT | 65°C, 30 s | 72°C, 30 s | 357 bp |
| *Ubqln5* | WT | TAGGTGAAAGGACATCACCG | CGGTCAGCGTTACAGTGAAGG | 65°C, 30 s | 72°C, 30 s | 315 bp |
|  | KO | TAGGTGAAAGGACATCACCG | CGGTCAGCGTTACAGTGAAGG | 65°C, 30 s | 72°C, 30 s | 307 bp |
| *Usp26* | WT | ACTCCGAGATTGTTAATCAGTATACG | GGATGGAAGCAAAGACAACTCACG | 65°C, 30 s | 72°C, 30 s | 528 bp |
|  | KO | ACTCCGAGATTGTTAATCAGTATACG | GGATGGAAGCAAAGACAACTCACG | 65°C, 30 s | 72°C, 30 s | 527 bp |
| *Vwa3b* | WT | GCTGACAGCAAAGACAGAGCTG | CAGCACTATGGTGACTCTCTGTTC | 65°C, 30 s | 72°C, 30 s | 148 bp |
|  | KO | GCTGACAGCAAAGACAGAGCTG | CAGCACTATGGTGACTCTCTGTTC | 65°C, 30 s | 72°C, 30 s | 131 bp |

**Supplementary Table S4.** The 34 mouse genes knocked out in this study. The TPM and RPKM values indicate the gene expression levels in mouse testis. TPM values are provided by EST profile in NCBI’s UniGene database, while RPKM values are obtained from RNA profiling data generated by the Mouse ENCODE project. The RBRC No. and CARD ID are available for the mouse lines that have been deposited as frozen sperm to Riken BioResource Research Center and Center for Animal Resources and Development at Kumamoto University, respectively.

| **Gene symbol** | **Gene name** | **Testis expression** | | **RBRC No.** | **CARD ID** |
| --- | --- | --- | --- | --- | --- |
|  |  | **TPM** | **RPKM** |  |  |
| *1700001O22Rik* | RIKEN cDNA 1700001O22 gene | 426 | 131.878 | 09981 | 2536 |
| *1700010B08Rik* | RIKEN cDNA 1700010B08 gene | 229 | 142.165 | 09829 | 2449 |
| *Ankrd7* | Ankyrin repeat domain 7 | 188 | 1.69 | 09974 | 2529 |
| *Banf2* | Barrier to autointegration factor 2 | 82 | 47.805 | 10110 | 2583 |
| *Bpifa3* | BPI fold containing family A, member 3 | 73 | 69.402 | 10819 | 2799 |
| *Cct6b* | Chaperonin containing Tcp1, subunit 6b (zeta) | 221 | 23.644 | 10859 | 2838 |
| *Cwh43* | Cell wall biogenesis 43 C-terminal homolog | 0 | 1.248 | 09956 | 2511 |
| *Fam221b* | Family with sequence similarity 221, member B | 344 | 93.299 | 10841 | 2821 |
| *Fndc8* | Fibronectin type III domain containing 8 | 41 | 49.8 | 09826 | 2446 |
| *Gsg1* | Germ cell associated 1 | 894 | 1505.61 | 10822 | 2802 |
| *Hmgb4* | High-mobility group box 4 | 1173 | N/A | 10121 | 2594 |
| *Hyal6* | Hyaluronoglucosaminidase 6 | 82 | 6.112 | 09953 | 2508 |
| *Kdm5d* | Lysine (K)-specific demethylase 5D | 16 | 0.32 | 09779 | 2426 |
| *Mgat4d* | MGAT4 family, member C | 24 | 6.408 | 10860 | 2839 |
| *Morn3* | MORN repeat containing 3 | 139 | 68.493 | 10810 | 2790 |
| *Nudt10* | Nudix (nucleoside diphosphate linked moiety X)-type motif 10 | 0 | 0.489 | - | - |
| *Oxct2a* | 3-Oxoacid CoA transferase 2A | 32 | N/A | 10349 | 2719 |
| *Oxct2b* | 3-Oxoacid CoA transferase 2B | 139 | N/A |  |  |
| *Scp2d1* | SCP2 sterol-binding domain containing 1 | 287 | N/A | 10861 | 2840 |
| *Slc36a3* | Solute carrier family 36 (proton/amino acid symporter), member 3 | 41 | 33.255 | 10820 | 2800 |
| *Tex13a* | Testis expressed 13A | 24 | 19.37 | 10359 | 2729 |
| *Tex13b* | Testis expressed 13B | 8 | 0.577 | 10835 | 2815 |
| *Tex35* | Testis expressed 35 | 155 | 77.283 | 10358 | 2728 |
| *Tgif2lx1* | TGFB-induced factor homeobox 2-like, X-linked 1 | 24 | N/A | 10824 | 2804 |
| *Tktl2* | Transketolase-like 2 | 32 | 6.999 | 10862 | 2841 |
| *Tmem19* | Transmembrane protein 19 | 65 | 1.855 | 09853 | 2473 |
| *Tmem202* | Transmembrane protein 202 | 41 | 24.286 | 10839 | 2819 |
| *Tmem270* | Transmembrane protein 270 | 8 | 99.853 | 10863 | 2842 |
| *Trim17* | Tripartite motif containing 17 | 90 | 63.416 | 10864 | 2843 |
| *Trpd52l3* | Tumor protein D52-like 3 | 49 | N/A | 10811 | 2791 |
| *Ube2d2b* | Ubiquitin-conjugating enzyme E2D 2B | 197 | N/A | 09824 | 2444 |
| *Ubqln5* | Ubiquilin 5 | 49 | N/A | 09838 | 2458 |
| *Usp26* | Ubiquitin specific peptidase 26 | 32 | 0.835 | 10823 | 2803 |
| *Vwa3b* | Von Willebrand factor A domain containing 3B | 197 | 17.381 | 10865 | 2844 |

**Supplementary Table S5.** Additional information about the 34 mouse genes analyzed in this study. The presence of transmembrane (TM) domains and signal peptide is predicted using TMHMM and SignalP, respectively.

| **Gene symbol** | **NCBI UID** | **MGI ID** | **Chromosome** | **No. of TM domain** | **Signal peptide** | **Human orthologs** |
| --- | --- | --- | --- | --- | --- | --- |
| *1700001O22Rik* | 73598 | 1923631 | 2 | 0 | N | *C9ORF50* |
| *1700010B08Rik* | 75485 | 1922735 | 2 | 0 | N | *-* |
| *Ankrd7* | 75196 | 1922446 | 6 | 0 | N | *ANKRD7* |
| *Banf2* | 403171 | 2684961 | 2 | 0 | N | *BANF2* |
| *Bpifa3* | 73388 | 1920638 | 2 | 0 | Y | *BPIFA3* |
| *Cct6b* | 12467 | 1329013 | 11 | 0 | N | *CCT6B* |
| *Cwh43* | 231293 | 2444131 | 5 | 10 | N | *CWH43* |
| *Fam221b* | 242408 | 2441678 | 4 | 0 | N | *FAM221B* |
| *Fndc8* | 78919 | 1926169 | 11 | 0 | N | *FNDC8* |
| *Gsg1* | 14840 | 1194499 | 6 | 2 | N | *GSG1* |
| *Hmgb4* | 69317 | 1916567 | 4 | 0 | N | *HMGB4* |
| *Hyal6* | 74409 | 1921659 | 6 | 1 | Y | *-* |
| *Kdm5d* | 20592 | 99780 | Y | 0 | N | *KDM5D* |
| *Mgat4d* | 67555 | 1914805 | 8 | 1 | N | *MGAT4D* |
| *Morn3* | 74890 | 1922140 | 5 | 0 | N | *MORN3* |
| *Nudt10* | 102954 | 2147931 | X | 0 | N | *NUDT10* |
| *Oxct2a* | 64059 | 1891061 | 4 | 0 | N | *OXCT2* |
| *Oxct2b* | 353371 | 2664115 | 4 | 0 | Y | *OXCT2* |
| *Scp2d1* | 66328 | 1913578 | 2 | 0 | N | *SCP2D1* |
| *Slc36a3* | 215332 | 2665001 | 11 | 11 | N | *SLC36A3* |
| *Tex13a* | 67944 | 1915194 | X | 0 | N | *TEX13A* |
| *Tex13b* | 83555 | 1890544 | X | 0 | N | *TEX13B* |
| *Tex35* | 73435 | 1920685 | 1 | 0 | N | *TEX35* |
| *Tgif2lx1* | 245583 | 2387796 | X | 0 | N | *TGIF2LX* |
| *Tktl2* | 83553 | 1933244 | X | 0 | N | *TKTL2* |
| *Tmem19* | 67226 | 1914476 | 10 | 6 | N | *TMEM19* |
| *Tmem202* | 73893 | 1921143 | 9 | 4 | N | *TMEM202* |
| *Tmem270* | 76629 | 1923879 | 5 | 2 | N | *TMEM270* |
| *Trim17* | 56631 | 1861440 | 11 | 0 | N | *TRIM17* |
| *Trpd52l3* | 66745 | 1913995 | 19 | 0 | N | *TPD52L3* |
| *Ube2d2b* | 73318 | 1920568 | 5 | 0 | N | *-* |
| *Ubqln5* | 70980 | 1918230 | 7 | 0 | N | *-* |
| *Usp26* | 83563 | 1933247 | X | 0 | N | *USP26* |
| *Vwa3b* | 70853 | 1918103 | 1 | 0 | N | *VWA3B* |

**Supplementary Table S6.** Detailed genotype of CRISPR/Cas9-derived mutant mice determined by Sanger sequencing. Bases in uppercase and lowercase indicate exon and intron sequences, respectively.

| **Gene** | **Upstream sequence** (25 bp) | **Mutation** | **Downstream sequence** (25 bp) |
| --- | --- | --- | --- |
| *1700001O22Rik* | CTGACTCAGGATGTTCCGGCGCCAG | -1 | CCATGGAGGGGACCCCGGCCTACAA |
|  | TGACTCAGGATGTTCCGGCGCCAGT | +1 | CCATGGAGGGGACCCCGGCCTACAA |
| *1700010B08Rik* | AGAACCATGTCTGCCTGCATGCCAT | -3+19 (GAAACCTTTGAGGTTTCAG) | GTTTCCCGCCATGATGAAAATGGAC |
| *Ankrd7* | CCCACAGCCACTCCTCAGAAGTGCC | -8 | TGGCCAAAACCGCACCGCCCAGCCT |
| *Banf2* | tgtctgttgcagaagATGGACGACA | -17 | GCCTTTCTCTCTGAACCCATTGGGG |
| *Bpifa3* | CCAAAGACAGCTGTAAGCCCATCGA | -8110 | aggcatgattccatgagcaactggt |
| *Cct6b* | ggactgttgaaccATGGCTGCGATC | -44827 | GCTGGGATGTCTTCTCTTAGAGACT |
| *Cwh43* | tccgggtggtcgcagtcATGCCAGG | -7 | AGAGCAATTGCCCTGGAGACCTTGC |
| *Fam221b* | CATCTTGAAACACCTGCCTCCCTGA | -6315 | CCGGCGCTGGGAAGAACATGAGACG |
|  | CCATCTTGAAACACCTGCCTCCCTG | -6314+1 (T) | GACCGGCGCTGGGAAGAACATGAGA |
|  | CATCTTGAAACACCTGCCTCCCTGA | -6312 | CGACCGGCGCTGGGAAGAACATGAG |
| *Fndc8* | CCAGATGGCGACGGTATTCTGTAAA | -1 | TGGGGGGTGGAGAGGAGGCTGTACC |
| *Gsg1* | GCCACGTTGCAAGGCTCATGTCACC | -4636 | TCGGCGCTACAGGACAAAGAATTTC |
| *Hmgb4* | GGGGAAAAAGACCAGCTAAGGCCCA | -7 | TGTGTCTTCTTACATCCATTTTATG |
| *Hyal6* | ATTTAACATCATTCCGAATCCTTTC | -17 | CACTATTACCGTATTTTATCCAAAG |
| *Kdm5d* | TCTACCGCCGCCTGAGTGCCCTGTG | -14 | GGCGGAATTCCGCGATCCTCTTGGC |
| *Mgat4d* | ctcagtgcctgtgcagcctggtgtt | -36 | AACTTGCTCTTCGCCTTCGTTGCCG |
| *Morn3* | TGTTTGCTGTGAACGGCGATCACTA | -1+2 (TG) | GTGGGCGAATGGAAGGGCAACTTGA |
| *Nudt10* | GGATGAAGTGCAAGCCGAACCAGAC | -106 | CGCTGGATCGTGCCGGGAGGGGGCA |
| *Oxct2a* | GGCTTCTGGCCTGGGCGCTCCCACG | -1 | GGGGTATCTGCTCTCCGCCCACCAC |
| *Oxct2b* | TGGGCAGCAGGAATGGCCAAGGGTG | -1722 | ATTTTCCAGTTAGTGACAACTGGAC |
| *Scp2d1* | gATGTGGAAGAGACCTGATCCTCAA | -594 | tgtgtacctttgctgggatccggaa |
| *Slc36a3* | CAGCTAGGCACCAGAACTTCCGGTC | -26893 | aaaaggccaggcgaggccatgcatg |
| *Tex13a* | atggtaggcaggtctttgcacgggt | -1861 | gtgtgtgtgtgtgtgtgtgtgtgtg |
| *Tex13b* | AGCCCCTAGAGCACTCAGCCATCAT | -3340 | ctggctataaagtgtgttgagagga |
| *Tex35* | gacccatagctagcaacactctcgg | -9421 | CATGTGCTGCCGACCTAGCACAGCC |
| *Tgif2lx1* | AAGGTAGTCCGGAAGAAACCCAGGA | -80 | CGAGAGGAAACATGTTGCCATTGAA |
| *Tktl2* | CAGGTGCTTCAGGACGTGGCCAATC | -1748 | CTACTGGATATGTTTGGAATCAGTG |
| *Tmem19* | GTAGCTCTATTTTCCATGACAGATA | -29 | aagatgattaccaatatagtaatac |
| *Tmem202* | GAGGCTTGCACCTACATCCGGATGT | -6002 | AGTGGAGGGCTGGAGAAACGGCTCA |
|  | AATGAGGCTTGCACCTACATCCGGA | -6006 | GTGGAGGGCTGGAGAAACGGCTCAG |
|  | ATGAGGCTTGCACCTACATCCGGAT | -6003+7 (ATAAGAA) | GAGTGGAGGGCTGGAGAAACGGCTC |
| *Tmem270* | ggaggccagacATGGAGGCCGCTCC | -4985 | TCGGGGCCCCCGCCTCAGTTCTTAA |
| *Trim17* | GAAAGGGAAGAAGAAGCAGAAGGGC | -6312 | gtgccacagttccccagaaggcagg |
| *Trpd52l3* | CCCCATCAAAAGCCTCCACTCCTCT | -749 | GCCAAACCTGTATCTATGTATTCCT |
| *Ube2d2b* | AAGAGAATCCACAAGGAACTGAACG | -382 | GGAATGGACTCAGAAATATGCGATG |
| *Ubqln5* | ctcttcttcaggaaatATGGCACGA | -8 | AGAAGCAGGGGACAGCCAGCTGGTG |
| *Usp26* | CTTCAAACCTATGGCGAAGACCAGA | -1 | TTACCTACATTTGACTTTTAAAAAT |
| *Vwa3b* | CACAAGCCGTGGAGAGCTACAAGCA | -17 | CCAGCGGAAGCCGGCAGATTTTTGG |

**Supplementary Table S7.** Outcomes of fertility tests for the four ubiquitously expressed gene knockout mouse lines.

| **Gene symbol** | **Genotype** | **No. of males** | **No. of total pups** | **No. of total litters** | **Mating period** | **Average litter size ± SD** |
| --- | --- | --- | --- | --- | --- | --- |
| *Cwh43* | -7/-7 | 2 | 131 | 17 | 20 weeks | 7.7 ± 2.2 |
| *Kdm5d* | X/-14 | 2 | 72 | 11 | 15 weeks | 7.2 ± 2.3 |
| *Nudt10* | -106/Y | 2 | 43 | 6 | 9 weeks | 7.1 ± 2.8 |
| *Tmem19* | -29/-29 | 3 | 23 | 3 | 4 weeks | 7.6 ± 0.56 |

**Supplementary Figure S1.** Heat map showing the expression patterns of all 34 genes in multiple tissues and organs. Expression levels are based on the TPM values provided by NCBI’s UniGene database. According to UniGene, *Fndc8* has a higher expression level in joint compared with testis and *Tmem202* shows a stronger expression in oviduct than in testis.


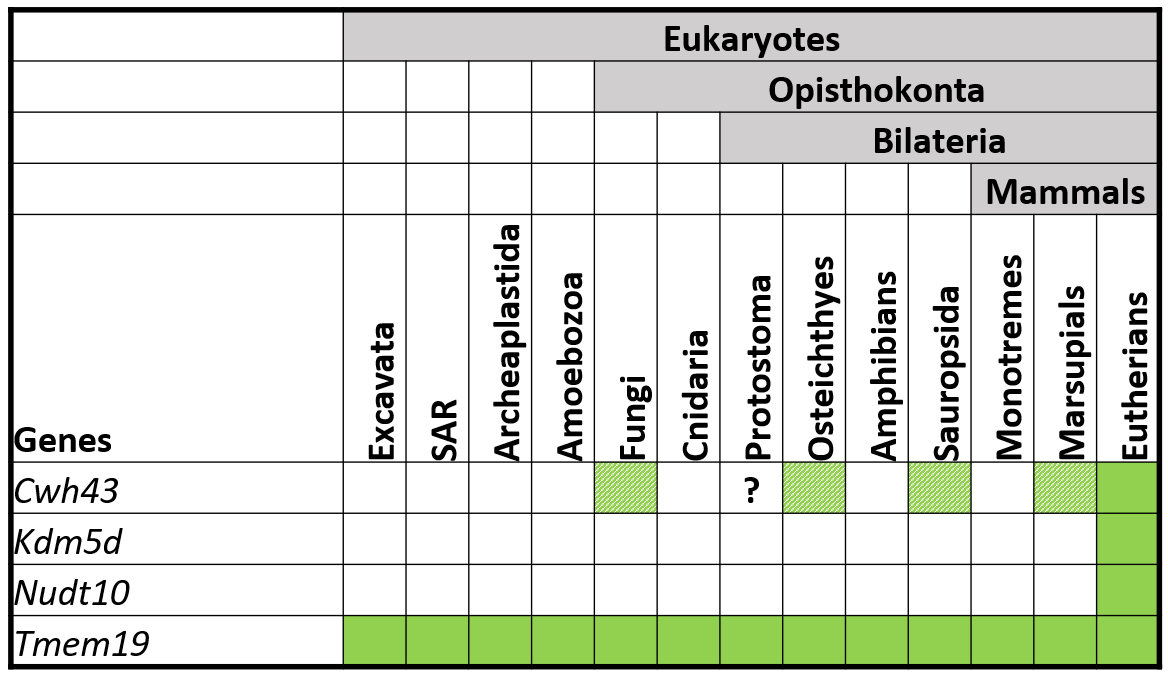


**Supplementary Figure S2.** Conservation of the four ubiquitously expressed genes among species. The presence of orthologs is indicated by colored or shaded squares. A gene conserved in all species in a taxon is highlighted with solid green. Shaded green indicates loss of ortholog in several species within a taxon. Question mark indicates potential orthologs in species within a taxon. *Tmem19* is presented in all taxa.


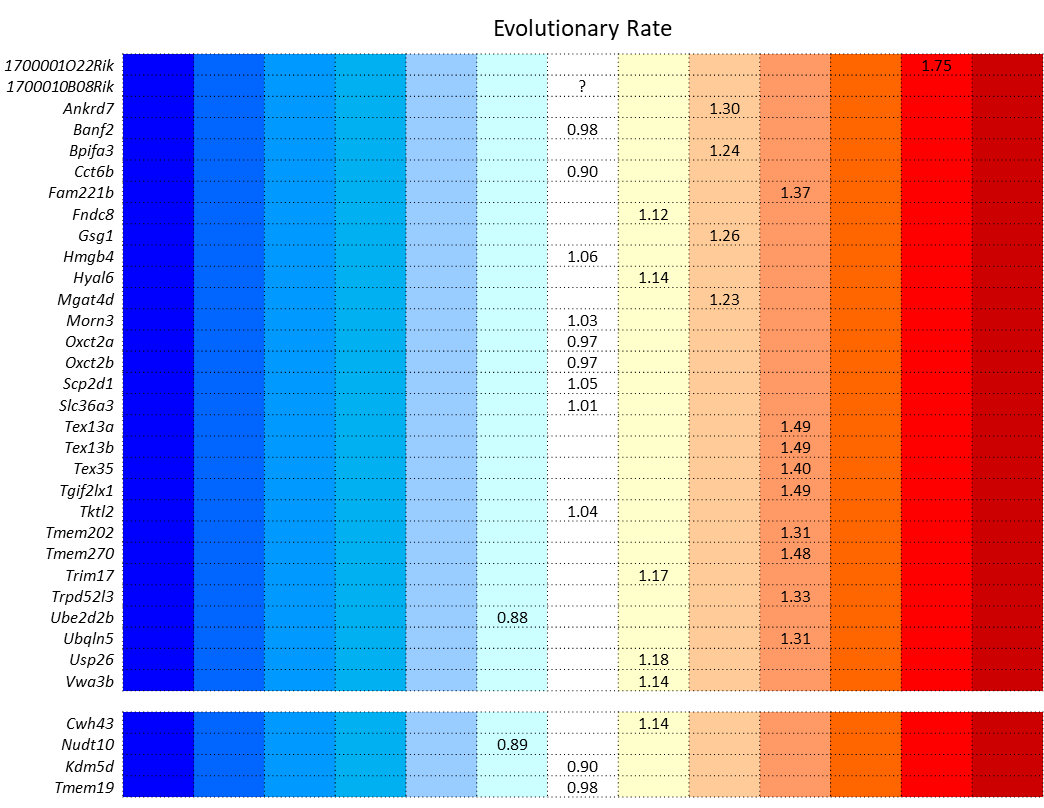


**Supplementary Figure S3.** Evolutionary rates of the 30 testis-enriched genes and 4 ubiquitously expressed genes predicted by OrthoDB. The evolutionary rate of *1700010B08Rik* is indicated by a question mark as this gene cannot be found in OrthoDB database.


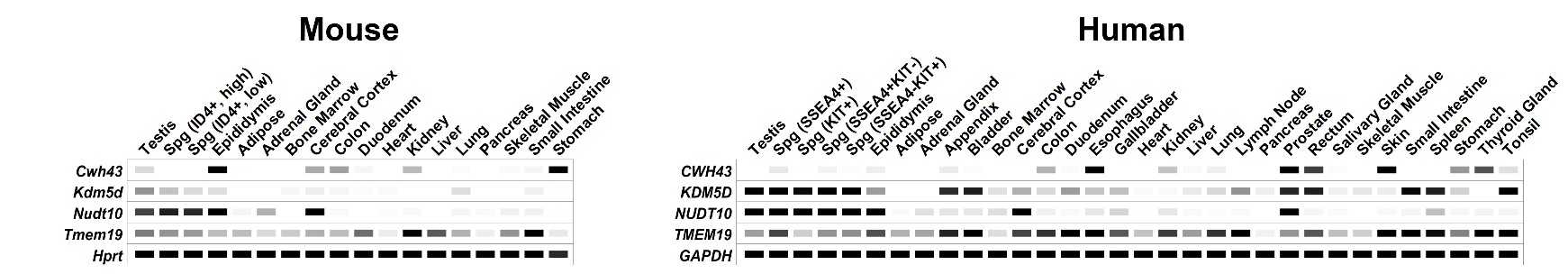


**B**

**A**

**Supplementary Figure S4.** The expression patterns of the 4 ubiquitously expressed genes in multiple tissues and organs revealed by digital RT-PCR. **(A)** The patterns of gene expression in mice. **(B)** The patterns of gene expression in human.

**A** *Hmgb4*^+/+^


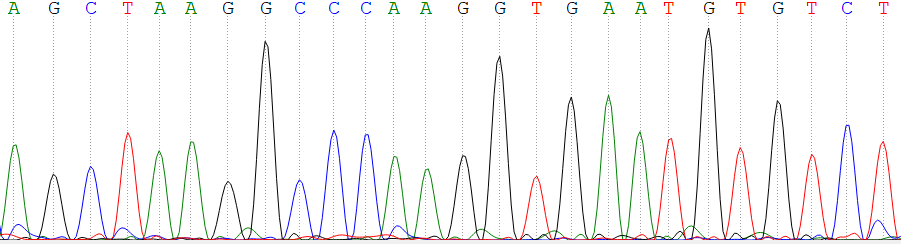


**B** *Hmgb4*^+/-7^


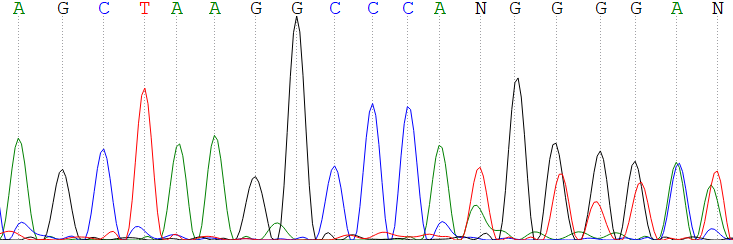


**C** *Hmgb4*^-7/-7^


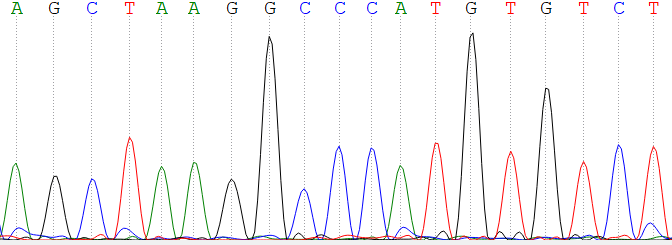


**Supplementary Figure S5.** Representative genotyping of knockout mice that were generated using the CRISPR/Cas9 system. Sanger sequencing of *Hmgb4* **(A)** wild-type, **(B)** heterozygous, and **(C)** homozygous mice. The deleted region is highlighted in red. Heterozygous deletion of *Hmgb4* in one allele results in overlapping waves.
